# Tri-functional T cell receptor antigen coupler (Tri-TAC): a novel method to direct T cells against tumors

**DOI:** 10.1186/2051-1426-2-S3-P17

**Published:** 2014-11-06

**Authors:** Christopher Helsen, Ben Li, Galina Denisova, Jonathan L Bramson

**Affiliations:** 1 McMaster University, Hamilton, Ontario, Canada

## 

Engineering T cells with chimeric antigen receptors (CARs) is proving to be an effective method for directing T cells to attack tumors in an MHC-independent manner. Current generation CARs aim to recapitulate T cell signaling by incorporating modular functional components of the TCR and costimulatory molecules. Development of next generation CARs has relied upon trial and error evaluation of signaling domains. We sought to develop an alternate method to re-direct the T cell receptor which does not rely upon the incorporation of signaling domains into the chimeric receptor. To this end, we developed a tri-functional molecule which is membrane-anchored and redirects the TCR in the presence of tumor antigen. We also included components of the CD4 co-receptor to provide requisite Lck signaling upon ligation of the tumor antigen. Our prototype receptor was directed against the HER-2 proto-oncogene. We have determined that engineering peripheral blood T cells with this novel receptor (termed a Tri-TAC) engenders tumor-antigen specific activation of numerous T cell functions, including cytokine production, degranulation and cytolysis - equivalent to, if not greater than, a 2nd generation CAR bearing the CD28 and CD3zeta signaling domains. Future iterations of the engineered T cells will include chimeric costimulatory receptors to enhance T cell functionality and reduce off target toxicity. This research was supported by the Canadian Institutes of Health Research and the Terry Fox Foundation.

